# Extra-Cellular Vesicles Derived from Thyroid Cancer Cells Promote the Epithelial to Mesenchymal Transition (EMT) and the Transfer of Malignant Phenotypes through Immune Mediated Mechanisms

**DOI:** 10.3390/ijms24032754

**Published:** 2023-02-01

**Authors:** Stefania Mardente, Michele Aventaggiato, Elena Splendiani, Emanuela Mari, Alessandra Zicari, Giuseppina Catanzaro, Agnese Po, Lucia Coppola, Marco Tafani

**Affiliations:** 1 Department of Experimental Medicine, Sapienza University of Rome, 00161 Rome, Italy; 2 Department of Molecular Medicine, Sapienza University of Rome, 00161 Rome, Italy

**Keywords:** extra-cellular vesicles, thyroid cancer, microRNAs, HMGB1, EMT

## Abstract

Thyroid cancer is the most common endocrine cancer, and its incidence is increasing in many countries around the world. Among thyroid cancers, the papillary thyroid cancer (PTC) histotype is particularly prevalent. A small percentage of papillary tumors is associated with metastases and aggressive behavior due to de-differentiation obtained through the epithelial–mesenchymal transition (EMT) by which epithelial thyroid cells acquire a fibroblast-like morphology, reduce cellular adhesion, increase motility and expression of mesenchymal proteins. The tumor microenvironment plays an important role in promoting an aggressive phenotype through hypoxia and the secretion of HMGB1 and other factors. Hypoxia has been shown to drastically change the tumor cell phenotype and has been associated with increasing metastatic and migratory behavior. Cells transfer information to neighboring cells or distant locations by releasing extracellular membrane vesicles (EVs) that contain key molecules, such as mRNAs, microRNAs (miRNAs), and proteins, that are able to modify protein expression in recipient cells. In this study, we investigated the potential role of EVs released by the anaplastic cancer cell line CAL-62 in inducing a malignant phenotype in a papillary cancer cell line (BCPAP).

## 1. Introduction

Thyroid cancers comprise well-differentiated thyroid cancers (DTCs), poorly differentiated thyroid cancers (PDTCs), anaplastic thyroid cancers (ATCs), and medullary thyroid cancers (MTCs). DTCs account for 90% of all thyroid cancers and have a relatively good prognosis [1,2,3]. These tumors maintain the typical differentiation characteristics of thyroid tissue such as the ability to capture iodine, synthesize thyroglobulin, and respond to the thyroid stimulating hormone (TSH). These phenotypic properties are lost by cancer cells passing from well-differentiated to poorly differentiated carcinomas up to anaplastic carcinomas which are the most undifferentiated. About 80–85% of patients respond well to traditional therapies, such as surgery, radioiodine treatment, and TSH suppressive therapy. The remaining 15–20% of patients experience recurrence or persistence of the disease, often associated with resistance to radioiodine treatment [4,5].

In the first steps of the de-differentiation process, epithelial cancer cells acquire mesenchymal markers in a process called epithelial–mesenchymal transition (EMT) [6,7,8,9,10]. 

EMT is the first step in the metastatic process [9] in which the expression of epithelial markers give way to mesenchymal markers that are controlled at the transcriptional level by several factors, i.e., Snail, Slug, Twist, ZEB 1, and ZEB 2 [11,12,13]. At the post-transcriptional level, EMT gene-related expression programs are influenced by mechanisms controlled by microRNAs (miRNAs) that modify the translation status of mRNAs by associating with them. MiRNAs have been identified as important modulators of cancer EMT by directly targeting EMT-inducing transcription factors, such as miR-200 family, miR-141, miR-429, miR-29b, and miR39a or by targeting E-cadherin transcripts, such as miR-9 and miR-661 [14].

During tumor growth and the acquisition of metastatic phenotypes, there is growing evidence that the tumor microenvironment (TME), composed of extracellular matrix and stromal cells, plays an important role in survival, growth, and invasion through a complex network in which cells communicate via the release of soluble factors that induce activation or suppression of intracellular pathways. Conventional signaling pathways include the secretion of molecules, such as HMGB1, VEGF, and EGF, that mediate direct cell-to-cell interaction.

A number of recent studies [15,16,17,18] have shown that cancer cells release extracellular vesicles (EVs) that carry complex biological information in the form of mRNAs, miRNAs, and proteins. These functionally active substances, packed into EVs, are transported to both neighboring and distant cells that uptake them [19]. There are differences between the protein and nucleic acid content of EVs derived from tumor cells versus those derived from normal cells, and many of the proteins and RNAs found in tumor-derived EVs are known for their role in cancer development and progression [20]. EVs are thus increasingly recognized as key players in a series of cellular processes linked to the pathogenesis and progression of cancer [21]. 

Although the underlying mechanisms are still unclear, malignant transformation appears to be associated with increased release of EVs, which contributes to the spread of the transformed phenotype by intercellular transfer of oncogenes or post-transcriptional regulators. Cancer cells and stromal cells exchange EVs carrying proteins and nucleic acids that can affect the function of recipient cells [22]. EVs derived from cancer cells may contribute to the spread of the transformed phenotype and to the ability of tumors to escape immune surveillance. EVs derived from stromal cells, such as fibroblast cells and immune system cells, can affect cancer cell motility [23]. Tumor-derived EVs also stimulate endothelial angiogenic responses, enter the circulatory system and reach distant organs, where metastases are promoted. All this data suggests that EV-mediated crosstalk between tumor and stromal cells plays a significant role in cancer progression [24]. 

The content of EVs is crucial for cell-to-cell signaling. HMGB1, a nuclear protein that intervenes in the communication between tumor cells and the immune system [25], is a damage-associated molecule (DAMP) that is released during stress or hypoxia and acts as an alarmin or a cytokine able to relay signals of danger to immune cells [26]. More specifically, HMGB1 induces the NF-kB signaling pathway promoting tumor-related inflammation that converts the microenvironment into a permissive and favorable setting for tumor progression, inducing cell proliferation and migration [27]. When HMGB1 migrates from the cell nucleus to the cytoplasm, it interacts with the mitochondrial DNA and with several miRNAs [28]. In a previous study, we demonstrated that HMGB1, released by thyroid cancer cells, induces over-expression of miR-221/222 and that their overexpression is responsible for PTEN suppression [29]. This study focuses on the phenotypic changes induced by EVs derived from an anaplastic thyroid cancer cell line (CAL-62) and applied to a differentiated thyroid cancer cell line (BCPAP). 

## 2. Results

### 2.1. Isolation and Characterization of EVs Derived from CAL-62 Cells under Hypoxic and Normoxic Conditions

**Figure 1:**
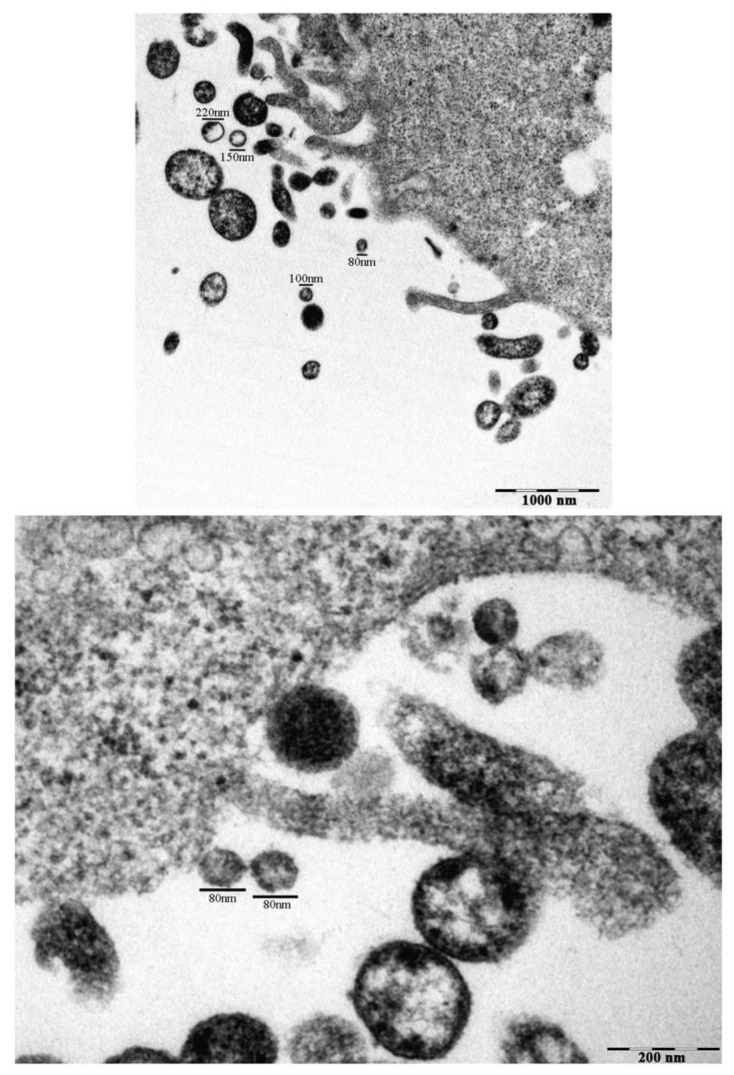
Extra-cellular microvesicles (EVs) in untreated CAL-62 cells observed by TEM. Transmission electron micrographs of CAL-62 cells with EVs, after 24 h culture with 5% FBS under normoxic conditions. Measurements of extra-cellular vesicles in the range of 40–200 nm are indicated (Bar: 100 µm).

Evaluation of CAL-62 cells and their immediate extracellular surroundings shows the presence of several EVs, ranging from 80 nm to 220 nm, as suggested by TEM (Figure 1).

We isolated and characterized EVs from CAL-62 cells under normoxic or hypoxic conditions for 24 h. No significant differences in the expression of CD81 were found between the two conditions (Supplementary Figure S1). 

### 2.2. EVs from CAL-62 Cells Contain HMGB1 and miRNAs and Induce Phenotypic Changes in BCPAP Cells

**Figure 2:**
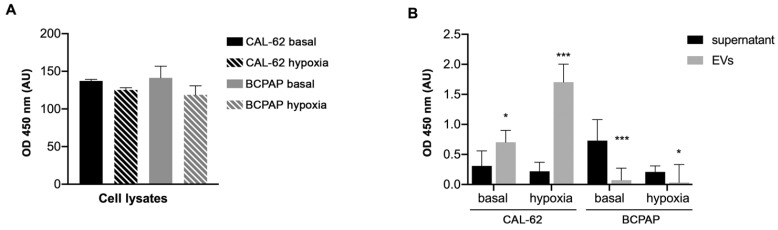
HMGB1 OD values in the different samples from CAL-62 cells and BCPAP cells cultured under normoxic and hypoxic conditions. ELISA analysis of (**A**) cell lysates, (**B**) supernatants and EVs lysates (100 µL/sample). *p* < 0.05 in populations indicated with asterisks (*). *p* < 0.005 in populations indicated with asterisks (***).

As HMGB1 is implicated in the progression of thyroid cancer [29] and has been shown to promote aggressive phenotypes in many tumors, we quantified its concentration in EVs and in supernatants from both CAL-62 and BCPAP cells. Figure 2 shows that HMGB1 release in EVs and supernatants differs quantitatively under the different culture conditions and in the different compartments that were evaluated. The difference in HMGB1 content between supernatants and EVs in CAL-62 is highly significant (*p* < 0.005). As HMGB1 is expressed in all eukaryotic cells, the amount of HMGB1 contained in cell lysates is approximately the same, while in supernatants and EVs it differs. This is in line with recent reports that demonstrate that the different roles of HMGB1 are related to its different redox forms and modes of release [30,31]. 

We then investigated miRNAs levels in EVs from CAL-62 and focused on miR-9, miR-200a and miR-200c because they are involved in EMT [32,33] and on miR-221 and miR-222, as we previously described their oncogenic role in papillary thyroid cancer cells [34].

**Figure 3:**
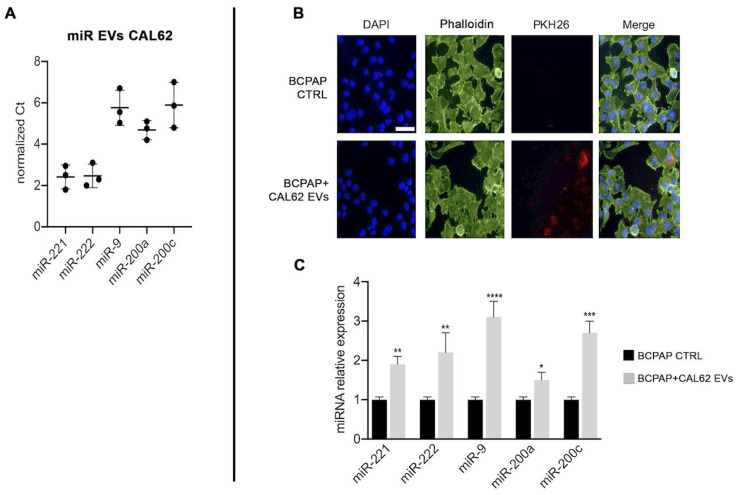
CAL-62 cell-derived EVs content and impact on BCPAP cells. Left Panel (**A**) miRNAs expression in EVs extracted from CAL-62 cell supernatants, normalized with U6. Right panel (**B**) confocal microscopy images of CAL-62 EVs internalized by BCPAP cells. Upper lane: control BCPAP cells. Lower lane: BCPAP cells treated with CAL-62 EVs. EVs labeled with PKH26 (red), nuclei stained with DAPI (blue), and cytoplasmic actin stained with phalloidin (green). Internalization reached its maximum after 2 h of exposure. Bar, 48.5 μm. (**C**) Relative expression of miRNAs normalized with U6. Asterisks indicate *p* values * *p* < 0.05, ** *p* < 0.01, *** *p* < 0.005, and **** *p* < 0.0001.

Figure 3A shows that all miRs investigated are expressed in CAL-62 EVs. Figure 3B,C shows the uptake of CAL-62-EVs into BCPAP cells after 2 h conditioning and the increased expression of miRNAs in relation to invasiveness and promotion of EMT after 24 h of CAL-62-EVs conditioning.

**Figure 4:**
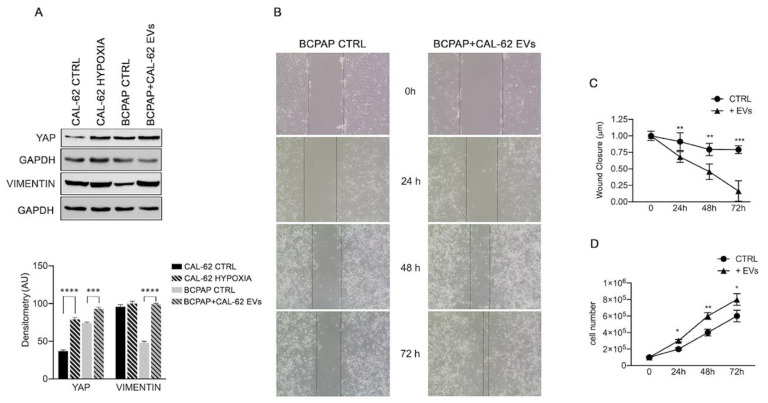
Effects of CAL-62 EVs on expression of EMT proteins in BCPAP cells (**A**) representative Western blot showing increase in YAP and vimentin in BCPAP cells treated with EVs from CAL-62 cells. Data are presented as mean ± s.d. from triplicate experiments. (**B**) Invasion test (indicative figure). (**C**) wound closure results of three independent experiments. (**D**) Proliferation rate increases in BCPAP cells treated with CAL-62 EVs. Asterisks indicate *p* values * *p* < 0.05, ** *p* < 0.01, *** *p* < 0.005, and **** *p* < 0.0001.

The EMT proteins YAP and vimentin also increased in BCPAP cells treated with CAL-62 EVs (Figure 4A). The acquisition of a “hypoxic state” by CAL-62 cells was confirmed by the HIF1α increase (Supplementary Figure S2).

Interestingly, HIF1α increase was also observed in normoxic BCPAP cells after treatment with EVs from CAL-62 (Supplementary Figure S2). However, we did not observe an increase in HIF1α metabolic targets such as hexokinase II (HKII) or carbonic anhydrase IX (CAIX), or the DAMP receptor RAGE (Supplementary Figure S2). The functional studies, shown in Figure 4, demonstrated that, when compared with untreated controls, the wound width rapidly decreased (Figure 4B,C) and the rate of cell proliferation in BCPAP cells (Figure 4D) increased after the addition of CAL-62-EVs.

## 3. Discussion

Thyroid tumor-derived cell lines provide a good experimental model for studying the mechanisms underlying tumorigenesis and cancer progression because they arise from the same cell type but exert different histological features, associated with different biological behavior and degree of differentiation [35]. Thyroid tumors in vivo are usually infiltrated by different cells, including stromal cells and immune cells that are crucial for tumor progression and acquisition of aggressive phenotypes [36]. Knowledge of the pathways that induce cells to change their behavior is important for the identification of new therapeutic strategies that underpin immunotherapy. Soluble mediators secreted by both tumor cells and local immune cells have been studied in many tumors. In the thyroid cancer immune network in particular [37], cytokines such as TGFβ [26] and HMGB1 [34] have been correlated with tumor invasiveness. 

We have shown here that the anaplastic thyroid cancer cell line CAL62 transfers an invasive phenotype to the differentiated cell line BCPAP through the release of EVs that contain miRNAs and proteins such as HMGB1. It is known that HMGB1 function is related to the redox status of three cysteines (C23, C45, C106) and that the disulfide-semi-oxidized state works as a cytokine promoting inflammation and tumor progression [38]. We have previously shown that HMGB1 increases the expression of oncogenic miR-221 and miR-222 in CAL-62 cells and BCPAP cells and in the present study, we show for the first time that HMGB1 is packed into exosomal vesicles and secreted in vitro by the anaplastic cell line CAL-62, probably in order to preserve its semi-oxidized status, which functions as a cytokine that induces angiogenesis and factors related to cancer progression and survival in hypoxic conditions [34].

The cargo in EVs secreted in both hypoxic and normoxic conditions were similar and the addition of EVs obtained from CAL-62 cells cultured in both conditions led to the expression of known EMT proteins vimentin and YAP. 

HIF-1α, known to be induced in hypoxic conditions, was also induced in BCPAP cells treated with CAL-62 EVs, obtained under normoxic conditions (shown in Supplementary Figure S1). In this case, as already reported [39], it is possible that HMGB1 delivered into BCPAP cells by CAL-62 EVs induces HIF-1α. HIF-1α activation is correlated with metastasis and poor prognosis in many solid tumors [40,41]. HIF-1α activation triggers transcription of many genes (such as GLUT4, CAIX, HXII, and RAGE) that are involved in metabolic reprogramming and survival of cancer cells [42], as well as EMT genes such as TGF, NF-kB, and Notch [43,44]. Interestingly, our results show an increase in EMT-related proteins YAP and vimentin in BCPAP cells treated with CAL-62 EVs for 24 h. On the other hand, we did not detect an increase in CAIX, HXII, or RAGE (see Supplementary Figure S2). One hypothesis is that EMT induction is an early effect of EV treatment while reprogramming of metabolism requires a longer exposure time.

The message of migration and invasion is also delivered CAL-62 EVs. Notably, miRNAs that in our experimental model were found in EVs, are associated with many signaling pathways that are de-regulated in cancer, such as PI3K/AKT, JAK/STAT, NOTCH1, Ras, and ERK. Some miRNAs have been reported to target both translation of oncogenes and oncosuppressor genes, while in several recent studies opposite functions have been reported for the same miRNA in different tumors. It would be well worth investigating whether this is due to the functional state of the cells or to the fact that some mutations in tumor cell DNA produce different targets for the same miRNAs. In the experimental model presented here, although miR-200a and miR-200c are overexpressed in CAL-62 cells, only miR-200c is found in EVs while in BCPAP cells treated with Evs, in contrast with their more aggressive phenotype, expression of both miR-200a and miR-200c slightly decreases, confirming their oncosuppressor role [45]. 

MiR-9 also has a dual role in different settings [33]. In one report, miR-9 is shown to suppress the oncogene BRAF in thyroid papillary tumors [33] and to reduce the growth of tumor cells when it is over-expressed. Conversely, we report here that miR-9 is part of CAL-62 EVs cargo and contributes to the induction of the EMT in BCPAP cells, in line with many recent studies that associate miR-9 with EMT in thyroid cancer [33] and resistance to therapy in breast cancer [46,47,48]. In glioblastoma, miR-9 is associated with increased tumorigenicity and migration, and in cervical squamous cell carcinoma with proliferation, invasion, and metastasis [46].

In conclusion, we report a novel mechanism through which different EMT effectors together with HMGB1 may promote a malignant phenotype in a differentiated and low aggressive thyroid cancer cell line. These results open up new perspectives on the treatment of thyroid cancers, particularly in relation to the design of immunotherapies.

## 4. Materials and Methods

### 4.1. Cell Cultures

The papillary thyroid cancer cell line BCPAP and the anaplastic thyroid cancer cell line CAL-62 were obtained from ATCC and cultured in RPMI 1640 medium (R0883; Sigma-Aldrich, Milan, Italy) supplemented with 10% fetal bovine serum (FBS) (Sigma-Aldrich, F9665), 2 mM glutamine (G7513; Sigma-Aldrich), 100 units/mL penicillin and 0.1 mg/mL streptomycin (P0781; Sigma-Aldrich). Cells were maintained at 37 °C with 5% CO_2_ in a humidified environment. Hypoxic conditions were achieved by incubating cells in a hypoxia modular incubator chamber (Billups-Rothenberg, Inc., San Diego, CA, USA) where a 1% oxygen mix was flushed in for 4 minutes according to the manufacturer’s instructions. 

BCPAP cells (10^5^ cells/mL) were treated for up to 72 h at 37 °C with CAL-62 EVs. At the end of incubation time, cells were washed three times with PBS and counted using trypan blue exclusion assay (Abcam, Cambridge, UK) in an automated cell counter (TC20TM automated cell counter, Bio-Rad, Stockholm, Sweden). The same protocol was used also for mRNA, miRNAs, and Western blot analysis.

### 4.2. EVs Isolation and Quantification from CAL-62 Supernatants

CAL-62 cells (90% confluence) were washed and cultured under hypoxic (1%) or normoxic conditions with serum-free RPMI supplemented with 2 mM glutamine, 100 units/mL penicillin, and 0.1 mg/mL streptomycin for 24 h. Culture medium was centrifuged at 4000× *g* for 20 min to remove debris and supernatants were ultracentrifuged for 90 min at 100,000× *g*, at 4 °C, in order to obtain EVs. Pellets were resuspended in 200 µL PBS. Total protein concentration of EVs extracted from 180 mL CAL-62 supernatants was 200 ± 16 µg. EVs concentration was quantified using Micro BCA™ Protein-Assay-Kit (Cat. N. 23,235, Thermo Fisher Scientific, Waltham, MA, USA). Concentration was calculated by measuring the absorbance at 562 nm and then interpolating it on a BSA standard control with a range between 0–200 µg/mL.

### 4.3. Scratch Assay

BCPAP cells were seeded into 12-well plates at a density of 1 × 10^5^ cells per well. After cells reached 100% confluency, the adherent cell layer was wounded by scraping two crossing perpendicular lines with a sterile 200 µL tip. After a wash with PBS to eliminate detached cells, fresh RPMI supplemented with 2% FBS was added to control cells while RPMI supplemented with 2% FBS and enriched with EVs isolated from CAL-62 was added to the remaining wells in order to evaluate the migration rate. Wounds were observed and photographed under a Zeiss IM35 microscope (Zeiss) and a digital camera (Nikon Digital Sight DS-L1) at 0, 24, 48, and 72 h after the scratch was made. The wound closure was the mean gap in µm of five randomly chosen fields and was measured using Nikon Digital Sight DS-L1 at 10× magnification.

### 4.4. Flow Cytometry Analysis

EV preparations were checked for purity by flow cytometry. Phenotype of EVs was analyzed using FITC conjugated monoclonal antibodies anti-CD81(1:100, Bio Legend, San Diego, CA, USA). Cells were incubated with anti-CD81 antibody for 15 min, washed with PBS, and analyzed in a cytofluorimeter (Beckman-Coulter CytoFlex) provided with a 15 mV argon laser tuned at 488 nm.

### 4.5. HMGB1 ELISA Assay

Levels of HMGB1 protein in supernatants, cells, and EV lysates were quantified using the commercial sandwich enzyme-linked immunosorbent HMGB1 (IBL International-Hamburg, Germany). Precision parameters of the ELISA test used had a highly sensitive range of 0.2–10 ng/mL, as reported by producers.

### 4.6. RNA Extraction and RT-qPCR

RNA from cell lines and EVs was obtained by automated Maxwell RSC-Promega extractor, using, respectively, the Maxwell RSC miRNA Tissue Kit miRNA (CAT # AS1460, Promega) and Maxwell RSC miRNA Plasma and Serum kit (CAT N.AS1680, Promega).

Retro-transcription for gene detection was performed using High-Capacity cDNA Reverse Transcription Kit. For miRNA, a miRNA-specific RT was carried out using TaqMan™ MicroRNA Reverse Transcription Kit. TaqMan Individual microRNA assays (Cat. N.4427975, Thermo Fisher Scientific) were used to assess expression of hsa-miR-200a (Assay ID:001011), hsa-miR-200c (Assay ID: 000505), of hsa-miR-9 (Assay ID: 000583), hsa-miR-222 (Assay ID: 002276), has-miR-221 (Assay ID: 000524) and U6 snRNA (Assay ID: 001973). TaqMan gene expression assays (Cat. N. 4331182, Thermo Fisher Scientific) were used to assess mRNA expression of HMGB1 (Assay ID: Hs01590761g1) and ACTB (Assay ID: Hs01060665_g1). U6 and ACTB were used as normalization controls. 

QPCR was performed on cDNA using Applied Biosystems ViiA 7 Real-Time PCR (Thermo Fisher Scientific, Waltham, MA, USA). The relative expression levels of miRNAs and mRNAs were calculated and quantified using the 2^ΔΔCq method after normalization for the expression of the controls. All procedures were performed according to the manufacturer’s instructions.

### 4.7. Immunofluorescence

BCPAP (2 × 10^5^) were plated on a coverslip and either left untreated or treated with EVs isolated from CAL-62. First, EVs from CAL-62 cells were labeled with the lipophilic dye PKH26 (Sigma Aldrich), following manufacturer’s instructions. Labeled EVs were then added to BCPAP for 2 h. After that, BCPAP cells were fixed and stained with phalloidin. Nuclei were counterstained with DAPI. Finally, samples were washed with PBS 3 times (5 min/wash), and coverslips were mounted in ProLong Diamond Antifade Mountant (Life Technologies, Thermo Fisher Scientific, Carlsbad, CA, USA). For the analysis of the immunofluorescence, cells were acquired with an FV1200 MPE laser scanning confocal microscope (Olympus, Tokyo, Japan).

### 4.8. Protein Extraction and Western Blot

Cell pellets were resuspended in lysis buffer [solution containing 50 mM Tris-Cl, 250 mM sodium chloride, 5 mM ethylenediaminetetraacetic acid (EDTA), 0.1% Triton^®^ X-100 and 0.1 mM dithiothreitol (DTT, D9163; Sigma Aldrich) plus 1 mM phenyl methyl sulfonyl fluoride (PMSF, 93482; Sigma Aldrich), Protease inhibitor cocktail (PI; P8340; Sigma-Aldrich), 1 mM sodium orthovanadate (Na_3_VO_4_, S6508; Sigma-Aldrich) and 10 mM sodium fluoride (NaF, 201154; Sigma-Aldrich). Samples were incubated on ice for 30 min and then centrifuged at 12,000× *g* for 10 min. Supernatants were collected and protein concentration was determined by the Bradford assay (Bio-Rad, Milan, Italy 500-0205). Whole cell lysates were heat denatured for 5 min and separated on 10% SDS-PAGE gels, run at 80 V (for the stacking gel) and 120 V (for the resolving gel). Proteins were transferred onto a 0.45 μm nitrocellulose membrane (162-0115, Bio-Rad) and the blotting membranes were blocked with 5% non-fat dry milk for 1 h at room temperature and then incubated with primary antibodies overnight at 4 °C. At the end of incubation time, membranes were washed with 0.1% Tween^®^ 20 (P9416; Sigma-Aldrich) in PBS (PBST) for 30 min at room temperature and incubated with the appropriate secondary antibody for 1 h at room temperature. The detection of the analyzed protein was assessed by enhanced chemiluminescence (Lite Ablot^®^ TURBO, EMP012001; EuroClone, Milan, Italy) detected by blue X-Ray film (Aurogene, AU1101, Rome, Italy). Densitometric analysis of the bands was performed using Image J Software v1.51 (NIH, Bethesda, MD, USA).

The following primary antibodies were used for Western blot analysis: GAPDH (sc-137279 Santa Cruz Biotechnology, Dallas, TX, USA), HXKII (sc-6521 Santa Cruz Biotechnology), RAGE (sc-8229 Santa Cruz Biotechnology), HIF1α (D2U3T Cell Signaling 14179S), CA-IX (NB 100-417 Novus Biologicals), vimentin (D21H3 Cell Signaling 5741S), YAP (sc-376830 (G-6) Santa Cruz Biotechnology). Peroxidase-conjugated Affini pure goat anti-rabbit IgG (H + L) (111-035-003) and peroxidase-conjugated Affini pure goat anti-mouse IgG (H + L) (115-035-062) were purchased from Jackson Immunoresearch Laboratories, Inc. (Ely, UK).

### 4.9. Transmission Electron Microscopy (TEM)

EVs were isolated from CAL-62 cultures and added to cell pellets. EVs and cell pellets were fixed overnight at 4 °C with 2.5% glutaraldehyde in 0.1 M phosphate buffer pH 7.3, washed six times in PBS, and then post-fixed in 1.33% osmium tetroxide in the same buffer for 1 h at room temperature. Samples were dehydrated in increasing graded steps of ethanol and embedded in epoxy resin (Embed 812, Electron Microscopy Science, Perth, Australia). The polymerization was performed at 60 °C for 24–48 h. Ultra-thin sections (60–70 nm) were cut on a Reichert-Jung, Buffalo, USA, ultramicrotome (Leica Microsystems, Wetzlar, Germany) and picked up on copper grids. Sections were post-stained with uranyl acetate and lead hydroxide and then analyzed using a Philips CM-10 Transmission Electron Microscopy (FEI, Hamilton, ON, Canada); micrographs were recorded on Kodak 4489 sheet film.

### 4.10. Statistics

Statistical analysis was carried out using Graph Pad Prism version 8.4. Data are expressed as means ± SD. Student’s *t*-test was used to determine significant differences at *p*-value of 0.05. Asterisks shown in figures indicate significant differences between cell populations exposed or unexposed to EVs.

## Supplementary Materials

The following supporting information can be downloaded at: https://www.mdpi.com/article/10.3390/ijms24032754/s1.
