# Supplementary material for: Extra-Cellular Vesicles Derived from Thyroid Cancer Cells Promote the Epithelial to Mesenchymal Transition (EMT) and the Transfer of Malignant Phenotypes through Immune Mediated Mechanisms
Source: Int J Mol Sci. 2023 Feb 1;24(3):2754. doi: 10.3390/ijms24032754 (PMC9917007; doi:10.3390/ijms24032754)
Supplement: Supplementary file 1 [file ijms-24-02754-s001.zip › ijms-2139206-supplementary.pdf]

Figure S1 supplementary

Cytofluorimetric characterization of exosomes.

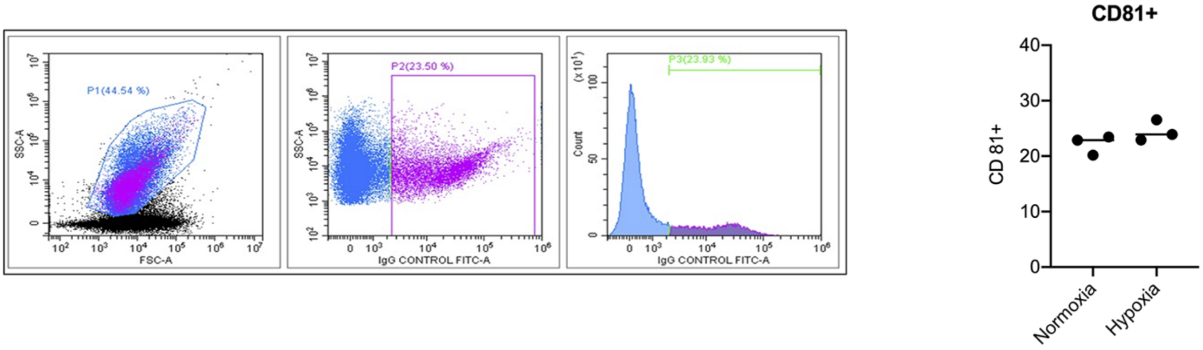

EVs obtained from CAL-62 supernatants, as described in Materials and methods, were labelled with FITC-CD81. Representative experiment showing that 24% of EVs were positive for CD81. Graph shows results obtained from 3 independent determinations.

Figure S2 supplementary

Expression of HIF 1α related proteins in CAL-62 cells and in BCPAP cells treated with CAL-62 EVs.

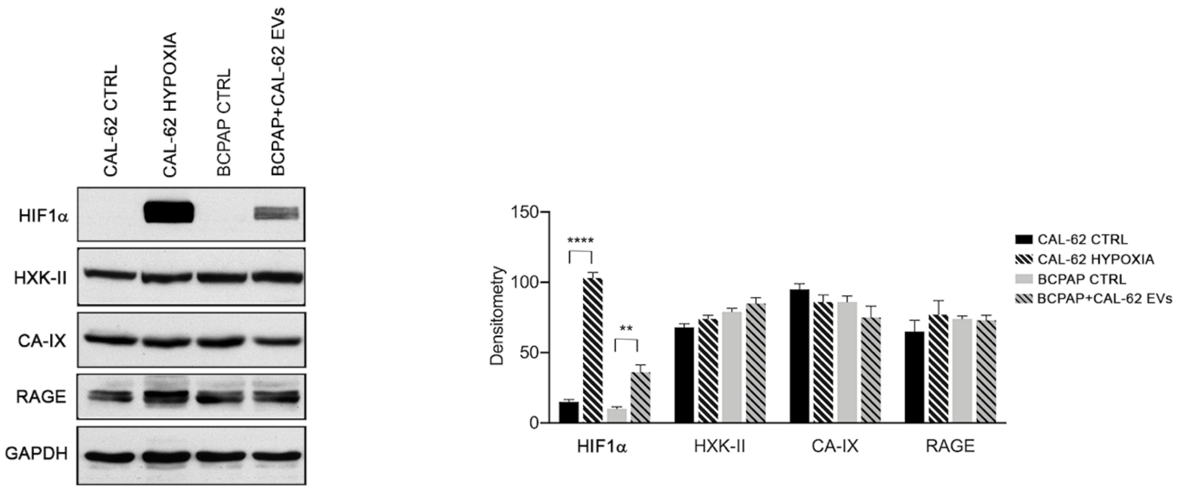

HIF 1α, HXK-II, CA IX and RAGE protein levels by western blot analysis, normalized with GADPH. In order to find out if the EMT phenotype was induced by HIF 1α even in normoxic conditions, we studied the expression of HXK, CAIX and RAGE that are induced during hypoxia (1). It is interesting to note that only HIF 1 α is induced in BCPAP cells treated with EVs obtained from CAL-62 cells cultured under hypoxic conditions and that none of the HIF 1α targets are induced in BCPAP cells after 24 h of CAL-62-EVs exposure. Asterisks indicate P values *P < 0.05, **P < 0.01, ***P < 0.005, and ****P < 0.0001. Data are presented as mean± s.d. from triplicate experiments

1. Masoud, G.N., Li, W. (2015). HIF-1α pathway: role, regulation and intervention for cancer therapy doi.org/10.1016/j.apsb.2015.05.007 2211-3835
